# Impact of Biochar on Douglas-Fir Tussock Moth (*Orgyia pseudotsugata* Lepidoptera: Erebidae) Larvae Reared on Synthetic Diet

**DOI:** 10.3390/insects12121065

**Published:** 2021-11-27

**Authors:** Stacey Rice-Marshall, Stephen P. Cook, John Randall

**Affiliations:** 1 Department of Entomology, Plant Pathology and Nematology, University of Idaho, 875 Perimeter Drive, Moscow, ID 83844-2329, USA; stephenc@uidaho.edu; 2 Department of Soil and Water Systems, University of Idaho, 875 Perimeter Drive, Moscow, ID 83844-2340, USA; jrandall@uidaho.edu

**Keywords:** forest defoliator, soil amendment, compensatory feeding

## Abstract

**Simple Summary:**

The novel use of carbon-rich biochar as a soil amendment in forest systems may be beneficial in the restoration of disturbed sites due to its ability to increase soil water holding capacity, potentially reduce drought stress in surrounding vegetation and aid in long-term carbon sequestration. As biochar is utilized in forest management, it is necessary to establish the potential effects that it may have on insects and other invertebrate assemblages. The results of recent laboratory studies demonstrate a potential for negative impacts on insects. Examining direct exposure of insects to biochar in a laboratory experiment may help us understand what effects biochar may have on insects that come into direct contact with the material. Along with direct exposure, biochar applications in the field would result in the surface and possible contamination of insect nutrient sources. To determine the impacts of ingesting biochar, we reared Douglas-fir tussock moth, *Orgyia pseudotsugata*, on synthetic diet to examine the insect’s survival and longevity.

**Abstract:**

The use of biochar as a soil amendment in forest ecosystems can be beneficial in the restoration of degraded soils. Forest insects such as the Douglas-fir tussock moth, *Orgyia pseudotsugata* (McDonnough) (Lepidoptera: Erebidae), may be exposed to biochar when the material is applied. Two experiments were conducted using biochar either (1) applied to the surface of the diet at three rates (0, 5, and 10 mg) or (2) incorporated into synthetic diet at four rates (0, 10, 20, and 40% volume/volume). The objective of both experiments was to determine if biochar on the surface or incorporated into a synthetic diet affected development and survival of *O. pseudotsugata* larvae. In both experiments, there was a significant decrease in estimated time to larval mortality in all biochar treatments compared to untreated controls. In the surface-applied biochar experiment, there was a significant difference in larval weight gain at day 12 between the control and 10 mg biochar treatments. In the experiment with biochar incorporated into the diet, mean larval weight at day 12 was highest in the low (10%) biochar treatment compared to all other treatments, although weight gain was only significantly different between the low- and high-concentration (40%) biochar treatments. Our results suggest that larvae, feeding on a low amount of biochar in the synthetic diet, may respond by engaging in compensatory feeding behavior. Fewer surviving larvae in the biochar treatment groups may contribute to the lack of significance found in the comparison of weight gain at day 24 in each experiment.

## 1. Introduction

Douglas-fir tussock moth (*Orgyia pseudotsugata* (McDunnough) (Lepidoptera: Erebidae) is a native forest insect in the western United States and Canada that primarily feeds on Douglas-fir (*Pseudotsuga menziesii* (Mirb.) Franco), true firs (*Abies* sp.) and spruce (*Picea* sp.) trees [1]. Each female moth may lay up to 200 eggs, all in a single egg mass, with the number of eggs per mass depending upon the phase of the infestation, as well as other environmental conditions [2]. Young larvae feed exclusively on new needles and, as the larvae mature, they switch to older, tougher needles. Past land management practices such as extensive timber harvesting and fire suppression have led to dense, overstocked stands that are dominated by shade-tolerant host species [3]. These stands are more susceptible to defoliator outbreaks than were presettlement forests [4]. Periodic outbreaks of *O. pseudotsugata* occur every 8 to 12 years when natural controls are unable to keep the population in check and can last 2 to 5 years, with defoliation contributing to growth loss, weakened trees, top-kill and tree mortality over large areas [5,6,7]. During an outbreak, suppression of *O. pseudotsugata* can be achieved with applications of chemical or microbial insecticides [5,8], bole-injected systemic insecticides [9], insect growth regulators [10], or mating disruption pheromones [7,11].

There is a complex of natural enemies associated with *O. pseudotsugata* including a naturally occurring nuclear polyhedrosis virus [12] as well as multiple parasitoids and predators [11,13,14]. In addition to these, the toxicity of monoterpenes and diterpene acids, which are present in host plant foliage, may also contribute to maintaining low population densities of insect herbivores [15]. Lockner et al. [16] reported that the direct exposure of five individual monoterpenes present in Douglas-fir (*Pseudotsuga menziesii* (Mirb.) Franco) foliage increased larval mortality of *O. pseudotsugata* larvae reared on synthetic diet. In addition to the existing natural enemies and occasional suppression techniques, a long-term management strategy to improve tree health may help protect susceptible forest stands against *O. pseudotsugata* and other defoliating insects. The application of biochar may improve overall tree resistance to defoliating insects and benefit tree growth at the same time. 

Biochar is a carbon-rich co-product of biomass pyrolysis [17], that is created in a high-temperature, low-oxygen environment [18]. Biochar can be made from any organic feedstock material such as woody residues, and could then be returned to the surface organic horizons [19,20,21]. Biochar has been used on forest [22,23] range [24], mine reclamation [25], and agricultural [20,26,27,28,29] soils as a method of improving greenhouse gas emissions [23,30], sequestering carbon [21] and improving soil properties [27,28,29,31,32]. Biochar may contribute to reducing plant herbivory by insect pests, either through physical contact with the material, or by improving overall plant resistance to herbivory. It may also be useful in priming the expression of plant defense-related genes [33], where improving overall plant resistance to herbivory may affect developmental and reproductive performances of the insect feeding on the plant [34].

*Orgyia pseudotsugata* larvae may come into contact with biochar on the foliage of the host tree, the surrounding understory vegetation and seedings, as well as the surrounding soil as larvae disperse between trees. Field observations indicate that larvae disperse with silk ballooning, or drop to the ground and crawl up to nearby trees and understory vegetation to find new foliage [1,2]. The effects of biochar in forest sites on herbivorous insects such as *O. pseudotsugata* has not been thoroughly examined. Cook and Rodrigues de Andrade Neto [35] noted a significant reduction in adult survival for three of the four insect species examined when they were in direct contact with dry biochar in confined arenas. *Formica obscuripes* (Forel) (Hymenoptera: Formicidae), *Ips pini* (Say) (Coleoptera: Curculionidae: Scolytinae) and *Temnochila chlorodia* (Mannerheim) (Coleoptera: Trogossitidae) had significantly reduced survival while *Enoclerus sphegeus* (Fabricius) (Coleoptera: Curculionidae: Scolytinae) did not. In addition, decreased survival and fecundity and increased duration of development has been reported for the brown rice planthopper, *Nilaparvata lugens* (Homopera: Delphacidae) reared in arenas with high concentrations of dry biochar [36].

Based on the potential to enhance soil carbon storage and to alter soil properties and processes with the addition of biochar on forest sites, we hypothesized that biochar would impact forest insects by altering their development and survival after ingestion. Therefore, we conducted two feeding trial experiments with the objective of determining if biochar either added to the surface, or incorporated into a synthetic diet altered the development and survival of *O. pseudotsugata* larvae in a controlled environment.

## 2. Materials and Methods

### 2.1. Insects and Synthetic Diet

A total of 374 *O. pseudotsugata* larvae from five egg masses were used in two feeding trial experiments. The egg masses used were collected in October 2017 from Douglas-fir (*Pseudotsuga menziesii* (Mirb.) Franco) and grand fir (*Abies grandis* (Dougl. ex D. Don) Lindl.) from two infestations in Packer John State Forest (44.18199, −116.04811 and 44.18450, −116.07502), approximately 93 km north of Boise, ID. Egg masses were maintained from October until April 2018 in a protected, outdoor enclosure in Moscow, ID (USA) where temperatures ranged from −6 °C to 14 °C. In May 2018, individual egg masses were affixed to the lids of approximately 475 mL clear plastic rearing containers with an approximate surface area of 56.75 cm^2^, with nylon mesh material glued over an opening in the lid for ventilation and filled approximately 25% with synthetic diet (Spruce Budworm Diet, Frontier Scientific Services, Newark, DE, USA). A 3 cm strip of Fluon^®^ Insect-a-slip insect barrier (BioQuip Products, Rancho Dominguez, CA, USA) was painted on the inside rim of the rearing container. Rearing containers were maintained at 22 °C in a 12:12 h (light:dark) regimen in environmental growth chambers (Percival Scientific, Perry, IA, USA). Only egg masses and larvae that appeared to be non-parasitized and disease-free were used. No parasitoids emerged from the selected egg masses, and no evidence of virus was observed. An open dish filled with deionized water provided humidity within each chamber.

Following egg hatch, larvae were reared under the same conditions as described above. Once larvae molted to the second instar, verified with discarded exuvia and observable true tufts (located at the prothorax and the first, second, and eighth abdominal segments) [2], they were removed from rearing containers, weighed, and randomly assigned to experiment and treatment (described below) with larvae from each egg mass evenly distributed among all treatments. One larva was placed in the appropriate container (approximately 60 mL plastic cups with cardstock lids) and maintained under the conditions described above for the duration of the experiment. In each experiment and biochar-diet combination, the diet (with appropriate treatment) was replaced as necessary due to desiccation, appearance of mold, or larval consumption.

In each experiment, larvae that appeared to be unhealthy at the start of the trial were eliminated from the experiment, leading to uneven sample sizes. For the duration of each experiment, larval survival was monitored daily, with mortality assigned to larvae that did not move or respond to stimulation with a paintbrush. Surviving larvae were weighed on days 12 and 24. Assays were conducted from June to July 2018. 

### 2.2. Biochar

Biochar used in this experiment was produced by pyrolysis of mixed conifer sawmill residues (primarily Douglas-fir and lodgepole pine (*Pinus contorta* Douglas ex Loudon)) in a gasification system (Tucker Engineering Associates, Locust, NC, USA). After gasification, the biochar was characterized by Anderson et al. as: pH = 10.2, moisture content = 2.94%, bulk density (dry) = 0.165 Mg m^−3^, carbon = 91.5%, nitrogen = 0.89%, C:N = 103.0, BET surface area = 15.0 m^2^ g^−1^, energy = 33.98 MJ kg^−1^ and a particle size distribution of <44 µm to 6.35 mm, centered around 0.84 mm [37]. Biochar was dried for 48 h at 35 °C to remove residual moisture before being ground and sifted into ≤105 µm sized particles using metal mesh particle sieves.

### 2.3. Experiment 1: Surface-Applied Biochar Treatments

*Orgyia pseudotsugata* larvae were reared on synthetic diet (Spruce Budworm Diet, Frontier Scientific Services, Newark, DE, USA) with dry biochar placed onto the surface (approximate surface area of 12.57 cm^2^). After initial weights were measured at the second instar stage, individual larvae were randomly assigned to diet-biochar treatment containers with 53, 57 and 51 larvae examined in the 0, 5 and 10 mg treatments, respectively (total sample size = 161).

### 2.4. Experiment 2: Incorporated Biochar Treatments

*Orgyia pseudotsugata* larvae were reared on synthetic diet (Spruce Budworm Diet, Frontier Scientific Services, Newark, DE, USA) with biochar uniformly mixed throughout the diet. Biochar at 0, 10, 20 and 40% (volume/volume) of the diet mixture was incorporated into the diet prior to being poured into containers. After initial weights were measured at the second instar stage, individual larvae were randomly selected and placed on the various diet-biochar mixtures, with 54, 53, 55 and 51 larvae examined in the 0, 10, 20 and 40% biochar treatments, respectively (total sample size = 213).

### 2.5. Statistical Analysis

All statistical analysis was conducted using SAS 9.4 analytical software [38]. Probit analysis was conducted for each experiment (biochar either on surface of, or incorporated into diet) and each treatment level to obtain lethal time (LT) estimates for 50% (LT_50_) and 95% (LT_95_) larval mortality of each treatment level with 95% confidence intervals. Non-overlapping confidence intervals were used to determine significant differences (*p* < 0.05). Kaplan–Meier curve analyses with Log-Rank tests and Tukey–Kramer adjustment for multiple comparisons, were used to analyze differences (*p* < 0.05) in mean larval survivorship for each experiment. 

Comparisons of initial larval weight (at day 0) were made among individual egg masses. Larval weight gain for two time intervals was calculated by subtracting initial larval weight (at day 0) from the weight of the same individual at day 12, and subtracting larval weight at day 12 from the weight at day 24. Comparisons of initial larval weight among egg masses, as well as larval weight gain among treatments within both food and biochar experiments (surface or incorporated biochar with synthetic diet), were made using generalized mixed models procedures (PROC GLMMIX) with lognormal distribution and *p* < 0.05 used to determine significance.

## 3. Results

### 3.1. Experiment 1. Surface-Applied Biochar Treatments

Of the larvae that were reared on synthetic diet with dry biochar applied to the surface, 62.3% were alive at day 12 in the control (0 mg biochar) and 49.1% were alive at day 24. In the 5 mg biochar treatment, larval survival to day 12 was 47.4% and 19.3% were alive at day 24. In the highest treatment concentration (10 mg biochar), 35.3% of the larvae were alive at day 12 and 21.6% were alive at day 24. 

There was a significant decrease in time to mortality (*p* < 0.05) for larvae exposed to either the 5 or 10 mg treatments of surface-applied biochar compared with the 0 mg control (Table 1). However, time to mortality between the individuals exposed to the 5 mg and 10 mg treatments was not significantly different. 

**Figure 1:**
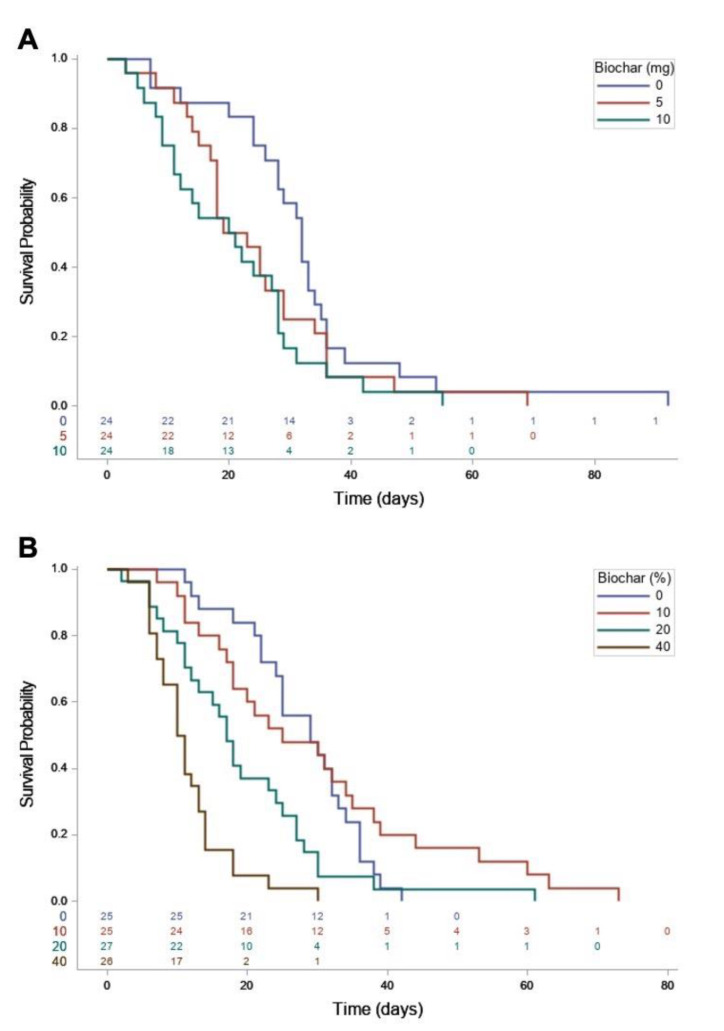
Kaplan–Meier survival curves for *O. pseudotsugata* larvae reared on (**A**) synthetic diet with dry biochar applied to the surface at three concentrations (0, 5 and 10 mg) and (**B**) biochar incorporated into synthetic diet at four rates (0, 10, 20 and 40% (by volume)) of the diet mixture. For each time interval, estimated survival percentage (survival probability), is calculated as the number of larvae surviving divided by the number of total larvae at the start of experiment for each treatment group. Counts of surviving larvae at each time point for each treatment are included beneath survival curves.

Analysis of Kaplan–Meier survival curves with multiple comparisons indicated differences in the survival of larvae exposed to 0 mg compared to 10 mg surface-applied biochar treatments (*p* = 0.0383). These comparisons showed control individuals lived longer on average than those feeding on biochar treated (both 5 mg and 10 mg) diet, although the difference in survival was not significant comparing the control and 5 mg biochar treatment (*p* = 0.2591) or the two surface-applied biochar treatments (*p* = 0.6866) (Figure 1).

**Figure 2:**
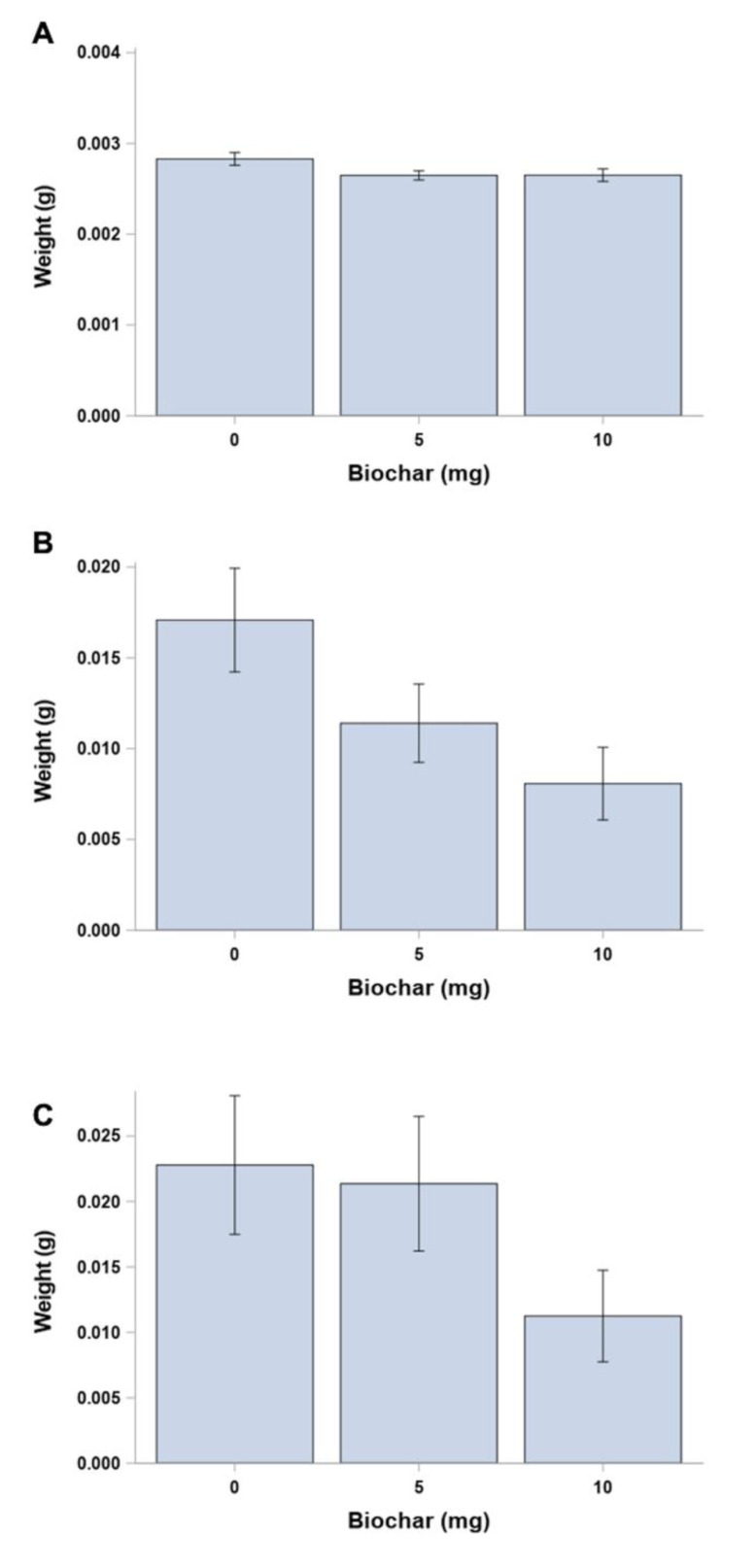
Mean larval weights (g ± SEM) of *O. pseudotsugata* larvae reared on synthetic diet with dry biochar applied to the surface at three concentrations (0, 5 and 10 mg). Initial weights were taken at (**A**) day 0 for all second instar larvae prior to randomly assignment to each treatment rate, then again at (**B**) day 12 and (**C**) day 24 for surviving larvae.

**Figure 3:**
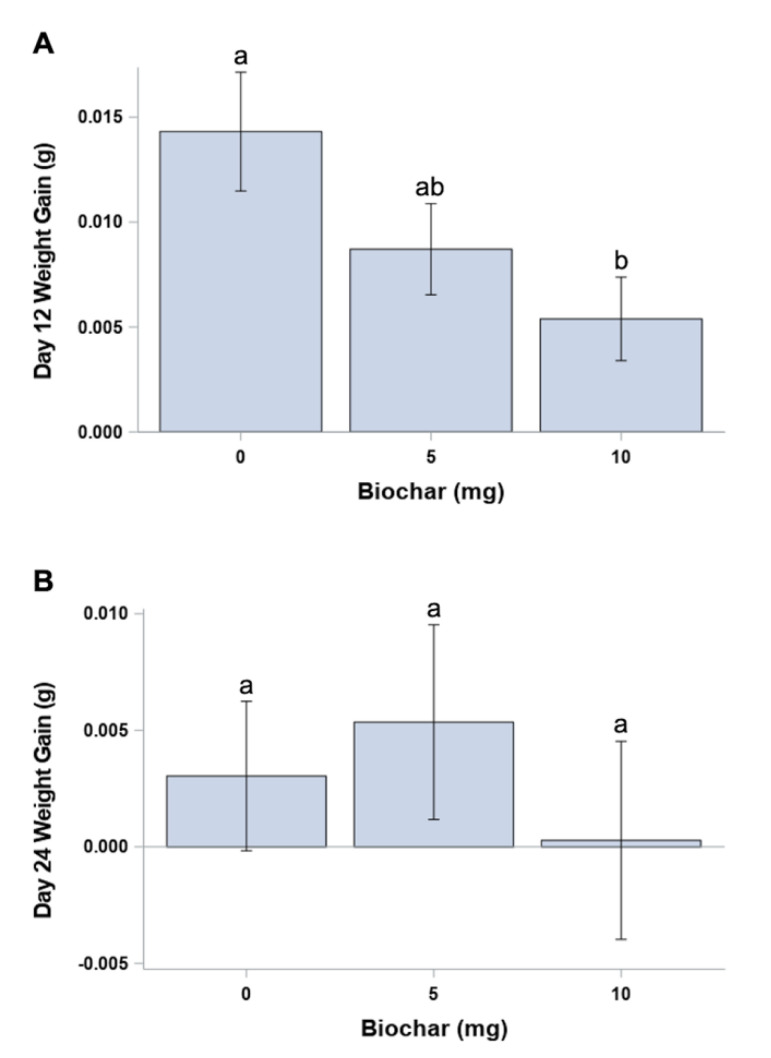
Mean (+ SEM) weight gain at 12 day intervals for individual larvae measured at (**A**) day 12 and (**B**) day 24 for *O. pseudotsugata* reared on synthetic diet with dry biochar applied to the diet surface at three concentrations (0, 5 and 10 mg). Means with the same letter are not significantly different (*p* < 0.05) based upon analysis of variance results.

Initial mean larval weights at day 0 were similar for all second instar larvae, independent of egg mass (*p* = 0.2013), prior to being randomly assigned to diet-surface biochar treatments (Figure 2A). Although mean larval weights appear to be similar among the treatments for each time point (Figure 2B,C), there was a significant difference (*p* = 0.0462) in larval weight gain from day 0 to day 12 (F= 2.32; *p* > F = 0.1069; df = 2, 60) between the control (0 mg biochar) and the treatment with the greatest amount of surface-applied biochar (10 mg biochar) (Figure 3A). However, there were no significant differences found when comparing larval weight gain between the control and low (5 mg biochar) treatments (*p* = 0.1762) or comparing the low- and high-concentration surface-applied biochar treatments (*p* = 0.4336) (Figure 3A). No significant difference was found in mean larval weight gain from day 12 to day 24 (F = 0.75; *p* > F = 0.4831; df = 2, 21) among all surface-applied biochar treatments (*p* > 0.25) (Figure 3B).

### 3.2. Experiment 2. Incorporated Biochar Treatments

Of the *O. pseudotsugata* larvae that were reared on biochar-incorporated diet, 66.7% were alive at day 12 in the control (0% biochar) and 46.3% were alive at day 24. Larval survival in the low concentration (10% biochar) treatment was 56.6% at day 12 and 32.1% at day 24. In the 20% biochar treatment, 50.9% of the larvae were alive at day 12 and 20.0% were alive at day 24. Furthermore, in the 40% incorporated biochar treatment, 25.5% were alive at day 12 and only 1.96% (one individual) was alive at day 24.

There was a significant decrease in time to mortality (*p* < 0.05) for larvae in each of the incorporated biochar treatments (10, 20 and 40%) compared to the untreated control (Table 2). Ingestion of the biochar material in the larval diet significantly decreased the lethal time estimation to 50% and 95% mortality as compared to the control, as each increase in the volume of biochar in the diet corresponded to a decreased time to mortality (Table 2).

Analysis of Kaplan–Meier survival curves with multiple comparisons showed a significantly greater probability of survival of control individuals compared to those feeding on 40% incorporated biochar treatment (*p* < 0.0001), as well as for larvae in 10% compared to 40% incorporated biochar treatments (*p* < 0.0001). Larval survival was also significant comparing the 10% and 20% incorporated biochar treatments (*p* = 0.0320). Overall, larvae in the 40% treatment showed the lowest probability of survival throughout the experiment. These comparisons showed control individuals lived longer on average than those feeding on biochar treated food, up until 24 days. Larvae in the 10% biochar treatment showed the greatest probability of survival after 24 days, even compared to the control individuals. The difference in larval survival was not significant comparing the control and the 10% biochar treatment (*p* = 0.9348), the control and 20% biochar treatment (*p* = 0.1571) or the two highest concentrations (20% and 40%) incorporated biochar treatments (*p* = 0.1299) (Figure 1).

**Figure 4:**
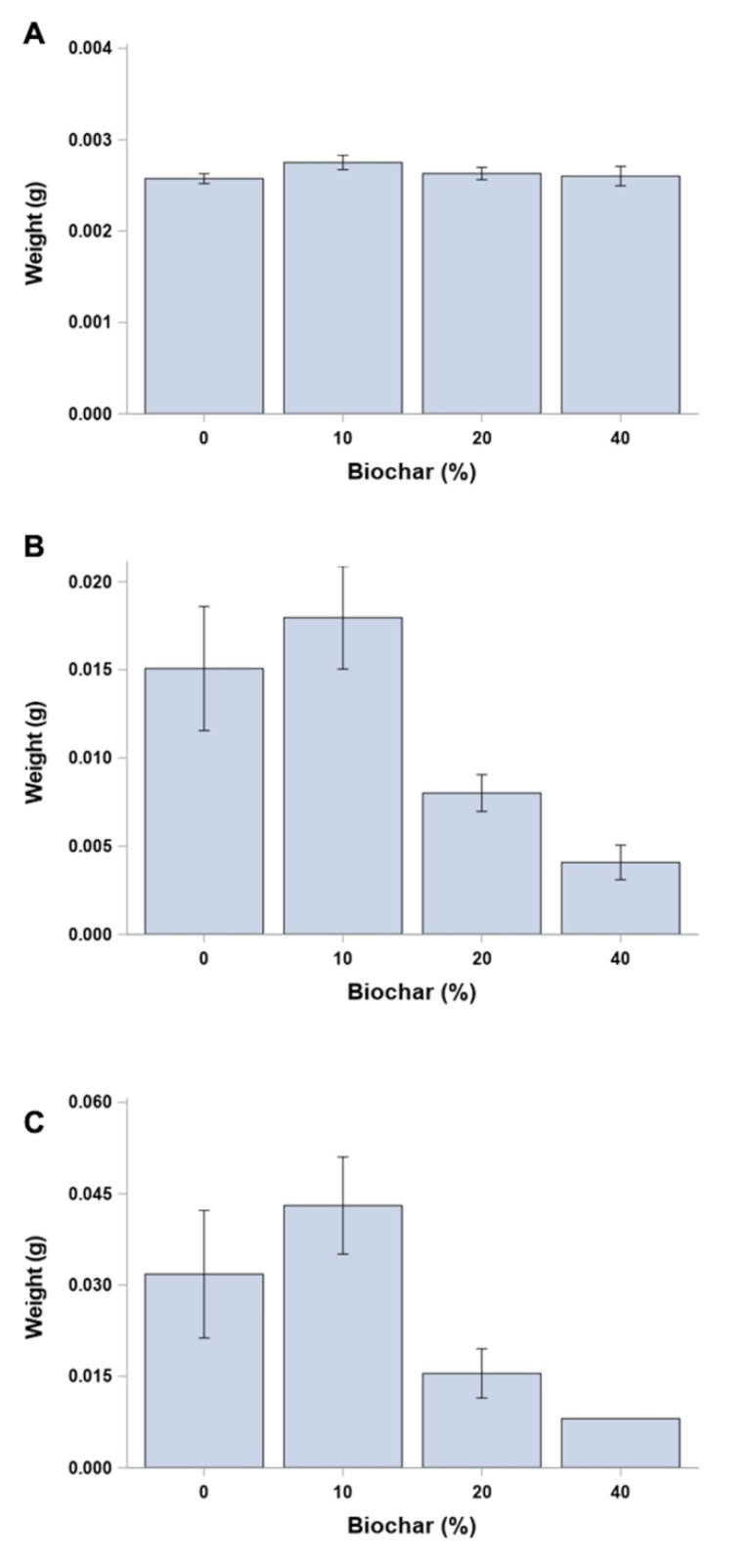
Mean larval weights (g ± SEM) of *O. pseudotsugata* larvae reared on synthetic diet with biochar incorporated into diet at four rates (0, 10, 20 and 40% (by volume)) of the diet mixture. Initial weights were taken at (**A**) day 0 for all second instar larvae prior to random assignment to each diet-biochar treatment, then again at (**B**) day 12 and (**C**) day 24 for surviving larvae. Only 1 larva survived to day 24.

**Figure 5:**
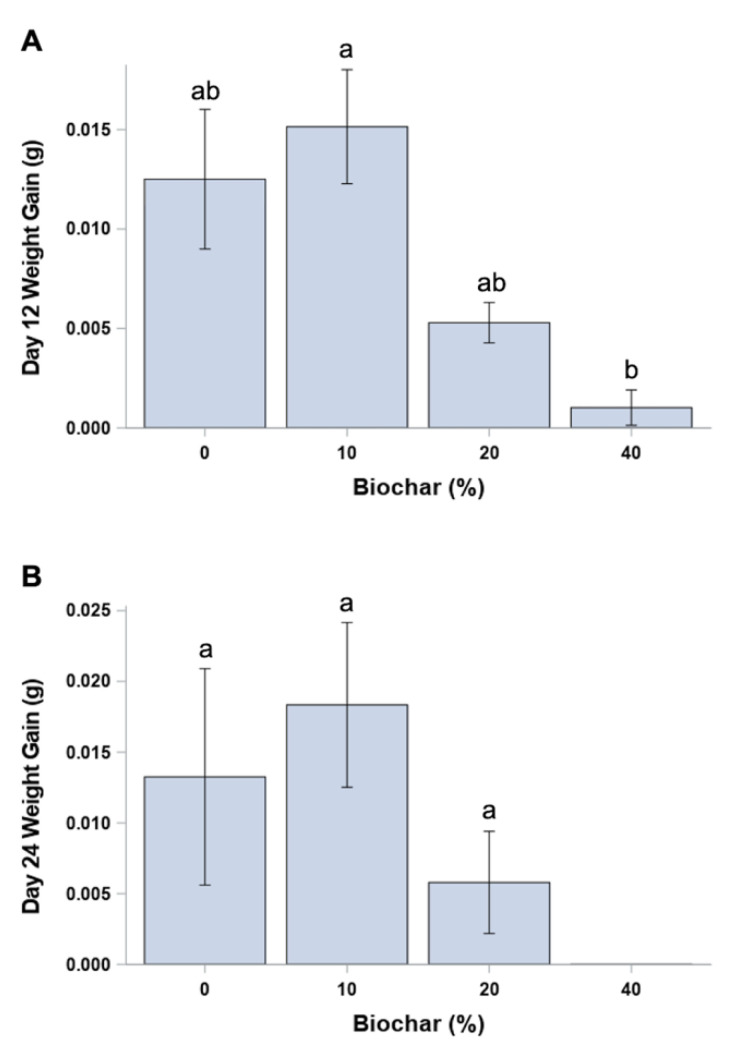
Mean (±SEM) weight gain at 12 day intervals for individual larvae measured at (**A**) day 12 and (**B**) day 24 for *O. pseudotsugata* reared on synthetic diet with biochar incorporated into diet at four diet:biochar (volume/volume) rates (0, 10, 20 and 40% biochar). Only 1 larva survived to day 24 and was not included in this analysis.

The average larval weight, independent of egg mass, at day 0 was similar, with no significant difference in weight among egg mass (*p* = 0.2800) prior to their random assignment to individual diet-biochar treatments (Figure 4). Overall, larval weight gain from day 0 to day 12 was highest in the low (10%) biochar treatment compared to all other treatments, although it was only significantly different (F = 1.61; *p* > F = 0.1932; df = 3, 82) between the low- (10%) and high-concentration (40%) biochar treatments (*p* = 0.0373) (Figure 5A). *p*-values are greater than 0.10 for all other comparisons of incorporated biochar treatments (Figure 5A). No significant difference in larval weight gain was found among the biochar treatments from day 12 to day 24 (*p* values greater than 0.39 for all comparisons) (Figure 5B). Fewer larvae survived to days 12 and 24 in each biochar-incorporated treatment compared to the control (0% biochar), with only a single larva surviving to day 24 in the highest (40%) biochar treatment.

The average larval weight gain from day 12 to day 24 was not significantly different (F = 1.61; *p* > F = 0.1932; df = 3, 82) among the control (0%), 10%, and 20% treatments (*p* > 0.39) (Figure 5B). The weight gain data for the single surviving larva in the highest concentration (40% biochar) treatment were not included in the analysis.

## 4. Discussion

Biochar material made from woody residues and applied as a soil amendment has been shown to improve soil health indices and is becoming a tool to sequester carbon on forest [22,23], range [24], mine reclamation [25], and agricultural sites [20,26,27,28,29]. In the laboratory, biochar can have a deleterious effect on survival [35] as well as development and fecundity [36] of insects that are directly exposed to the material in enclosed arenas. Further, studies on cereal grain pests have shown that in the laboratory biochar can decrease fertility and population growth [33], and these results in combination with our lab study show a potential for management of insect populations. However, field applications of biochar may impact insect populations and have not been thoroughly studied for forest insects that may be exposed to applied biochar. For example, freshly hatched *O. pseudotsugata* larvae descend from egg masses on silk strands and disperse with the wind to surrounding trees and understory vegetation where they begin to feed. Dispersing larvae may land on the ground and crawl to nearby vegetation [2,5,6,7]. When biochar is applied, a portion of the material becomes airborne and settles on foliage of trees and understory plants. Larvae would potentially be exposed to and ingest biochar if it were present on the foliage of trees and understory plants or on the soil surface. 

The biochar used in our study consisted of ≤105 µm sized particles, a small portion sifted from the manufactured biochar material. Efficient field application of biochar will likely consist of a mixture of particles ranging in size from large chunk to nano-particles, depending on the method of manufacture and application. Cook and Rodrigues de Andrade Neto [35] demonstrated a potential for negative impacts of insect species exposed to dry biochar material, with both fine (<150 mm) and coarse (>1.0 mm) particle sizes showing a decrease in survival. Moisture content as well as physical size of the biochar may affect how insects exposed to and potentially ingesting biochar may respond. 

In our first experiment, larvae were in direct contact with, and ingested biochar applied to the surface of the diet. Although we found statistically significant differences in the estimated time to mortality between the control treatment and each biochar treated group, by the end of this study, there was a reduced sample size which may have contributed to a lack of significance in time to mortality between the low- (5 mg) and high-concentration (10 mg) biochar treatments (Table 1). Fewer surviving larvae in the biochar treatment groups may also contribute to the lack of significance among all three treatments in experiment 1 when comparing larval weight gain from day 12 to day 24 (Figure 3). Further, several larvae were observed feeding restricted to a small area of the treatment cup, which may have been an attempt to avoid ingesting the biochar. However, larvae were exposed to biochar on the surface of the diet and therefore biochar was at least initially ingested prior to the larvae continuing to feed on diet underneath the surface layer of biochar. 

In the second experiment, larvae consumed the biochar which was uniformly incorporated into the diet. Our results suggest that the amount of biochar in the low (10%) treatment may result in larvae engaging in compensatory feeding behavior [39]. This behavior results in the consumption of more food of a lower quality in response to a decrease in dietary nutrients. The increased consumption may result in there being no decrease in mean weight compared with larvae reared on the control (0% biochar) diet. Several laboratory studies have shown that compensatory feeding occurs with insects fed with synthetic diets that are low in nitrogen or protein [39,40]. The high 103.0 C:N ratio for the biochar used in this study may possibly contribute to an overall lowered available N concentration in the diet, especially in those diets with biochar directly incorporated into the food. Addition of carbon in the form of biochar would alter or dilute the nutrients of the diet in a way that the larvae would need to consume more of it to reach similar stages of development compared to the diet with no biochar added (the control). A lower available N in the diet would therefore be consistent with a compensatory feeding theory. Additionally, the reduced sample size for the biochar-treated diets may have contributed to lack of significance among all four treatments comparing the weight gain of the larvae at day 24 (Figure 5B). 

**Figure 6:**
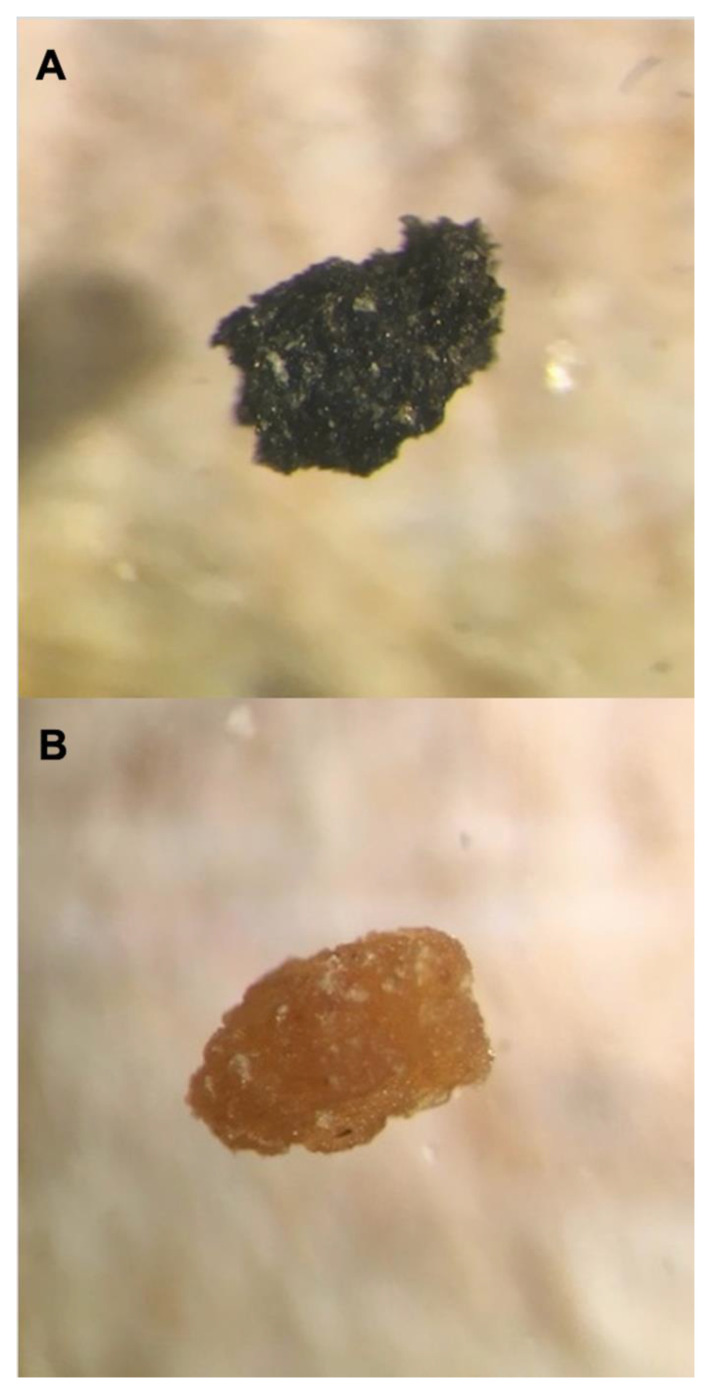
*Orgyia pseudotsugata* frass viewed at 40× magnification, collected from larvae reared on synthetic diet in (**A**) the 10% biochar treatment and (**B**) the control 0% biochar treatment.

Larvae were able to ingest and presumably pass biochar through their digestive tracts (Figure 6), although the physiological effects of the biochar, and specifically the mode of action, are unknown and should be addressed in future research. The alkaline pH and potential water holding capacity [21,37] of the porous biochar may affect the conditions of the larval digestive tracts and interrupt their ability to digest nutrients or absorb nutrients. Based upon the lower larval survival to 24 days and lower weights at each time point, it is possible that larvae reared on the biochar treated synthetic diet failed to develop normally. 

## 5. Conclusions

Our laboratory studies showed that the addition of biochar on herbivorous insect food sources had deleterious consequences on survival and weight gain of *O. pseudotsugata.* Biochar has the potential to impact tree resistance mechanisms, and the potential use of biochar applied to tree foliage and surrounding soil for suppression of herbivorous insects is undetermined. This work should be extended to long-term, large-scale field plots with known insect populations to assess the potentially suppressive effects of biochar. It may be possible that biochar will have direct impacts on insects ingesting the material, as well as improving overall tree health and ability to defend against insect herbivory.

## Figures and Tables

**Table 1:** Lethal time (LT) estimates to 50% (LT_50_) and 95% (LT_95_) mortality (maximum likelihood) with 95% confidence intervals (CI) for *O. pseudotsugata* larvae reared on synthetic diet with dry biochar material applied to the surface. Within a column, estimates of LT followed by the same letter are not significantly different.

mg Biochar	LT_50_ (days)	95% CI	LT_95_ (days)	95% CI
0	18.5 a	17.1–19.7	54.7 a	50.6–59.8
5	12.7 b	11.6–13.8	38.9 b	35.8–42.8
10	10.9 b	10.0–12.0	35.5 b	32.3–39.5

**Table 2:** Lethal time (LT) estimates to 50% and 95% mortality (maximum likelihood) with 95% confidence intervals (CI) for *O. pseudotsugata* larvae reared on synthetic diet mixed with multiple concentrations of dry biochar material by volume. Within a column, estimates of probability of mortality followed by the same letter are not significantly different.

(% Biochar)	LT_50_ (Days)	95% CI	LT_95_ (Days)	95% CI
0	18.7 a	17.6–19.8	42.0 a	39.2–45.5
10	16.2 b	14.9–17.4	51.9 b	47.8–57.0
20	12.4 c	11.3–13.4	36.6 c	31.8–38.1
40	8.9 d	8.1–9.6	19.0 d	17.3–21.4

## Data Availability

Data available on request.

## References

[1] Furniss R.L., Carolin V.M. (1977). Western Forest Insects. USDA Forest Service Miscellaneous Publication No. 1339.

[2] Mason R.R., Brookes M.H., Stark R.W., Campbell R.W. (1978). The Douglas-Fir Tussock Moth: A Synthesis. USDA Technical Bulletin 1585. Forest Service Science and Education Agency.

[3] Wickman B.E., Quigley T.M. (1992). Forest Health in the Blue Mountains: The Influence of Insects and Diseases. Forest Health in the Blue Mountains: Science Perspectives.

[4] Mutch R.W., Arno S.F., Brown J.K., Carlson C.E., Ottmar R.D., Peterson J.L., Quigley T.M. (1993). Forest Health in the Blue Mountains: A Management Strategy for Fire-Adapted Ecosystems. Forest Health in the Blue Mountains: Science Perspectives.

[5] Wickman B.E., Mason R.R., Trostle G.C. (1981). Douglas-Fir Tussock Moth.

[6] Kohler G. (2012). Douglas-Fir Tussock Moth (Orgyia pseudotsugata): Outbreak Status of a Conifer Defoliating Caterpillar.

[7] Pederson L., Eckberg T., Lowrey L., Bulaon B. (2020). Revised. Douglas-Fir Tussock Moth.

[8] McGrath D. (2001). Pacific Northwest Insect Management Handbook.

[9] Cook S.P., Sloniker B.D., Rust M.L. (2013). Efficacy of two bole-injected systemic insecticides for protecting Douglas-Fir from damage by Douglas-fir tussock moth and fir coneworm. West. J. Appl. For..

[10] Cook S.P. (2003). Laboratory and field evaluation of tebufenozide, diflubenzuron, and *Bacillus thurengiensis* var. kurstaki for suppression of Douglas-fir tussock moth (*Orgyia pseudotsugata* (McDunnough)) in Idaho: A case study. J. Econ. Entomol..

[11] Cook S., Wenz J., Ragenovich I., Reardon R., Randall C. (2005). Impact of mating disruption pheromone treatments to control Douglas-fir tussock moth, on egg parasitoids. Pan-Pac. Entomol..

[12] Vezina A., Peterman R.M. (1985). Tests of the role of a nuclear polyhedrosis virus in the population dynamics of its host, douglas-fir tussock moth, *Orgyia pseudotsugata* (Lepidoptera: Lymantriidae). Oecologia.

[13] Torgersen T.R., Mason R.R. (1987). Predation on Egg Masses of the Douglas-fir Tussock Moth (Lepidoptera: Lymantriidae). Environ. Entomol..

[14] Mason R., Torgersen T., Wickman B., Paul H. (1983). Natural Regulation of a Douglas-Fir Tussock Moth (Lepidoptera: Lymantriidae) Population in the Sierra Nevada. Environ. Entomol..

[15] Raffa K.F., Powell J.S. (2004). Tolerance of plant monoterpenes and diterpene acids by four species of Lymantriidae (Lepidoptera) exhibiting a range of feeding specificities. Great Lakes Entomol..

[16] Lockner A.D., Cook S.P., Kimsey M., McDonald A.G., Shaw T. (2019). Toxicity to Douglas-fir tussock moth and foliar concentration of individual monoterpenes in Douglas-fir foliage following fertilization in thinned stands. Northwest Sci..

[17] Biederman L.A., Harpole W.S. (2013). Biochar and its effects on plant productivity and nutrient cycling: A meta-analysis. GCB Bioenergy.

[18] Bridgewater A. (2004). Biomass fast pyrolysis. Therm. Sci..

[19] Abit S.M., Bolster C.H., Cai P., Walker S.L. (2012). Influence of feedstock and pyrolysis temperature of biochar amendments on transport of *Escherichia coli* in saturated and unsaturated soil. Environ. Sci. Technol..

[20] Lehmann J., Gaunt J., Rondon M. (2006). Bio-char Sequestration in Terrestrial Ecosystems—A Review. Mitig. Adapt. Strateg. Glob. Clim. Chang..

[21] Lehmann J., Joseph S. (2009). Biochar for Environmental Management: Science and Technology.

[22] Page-Dumroese D.S., Coleman M.D., Thomas S.C., Bruckman V., Varol E.A., Uzun Basak L.J. (2017). Opportunities and uses of biochar on forest sites in North America. Biochar: A Regional Supply Chain Approach in View of Climate Change Mitigation.

[23] Sarauer J., Page-Dumroese D.S., Coleman M.D. (2018). Soil greenhouse gas, carbon content, and tree growth response to biochar amendment in western United States forests. Glob. Chang. Biol. Bioenergy.

[24] Gao S., DeLuca T.H. (2020). Biochar alters nitrogen and phosphorus dynamics in a western rangeland ecosystem. Soil Biol. Biochem..

[25] Rodriguez-Franco C., Page-Dumroese D.S. (2021). Woody biochar potential for abandoned mine land restoration in the US: A review. Biochar.

[26] Glaser B., Lehmann J., Zech W. (2002). Ameliorating physical and chemical properties of highly weathered soils in the tropics with charcoal—A review. Biol. Fertil. Soils.

[27] Marris E. (2006). Putting the carbon back: Black is the new green. Nature.

[28] Waqas M., Khan A.L., Kang S.M., Kim Y.H., Lee J. (2014). Phytohormone-producing fungal endophytes and hard-wood-derived biochar interact to ameliorate heavy metal stress in soybeans. Biol. Fertil. Soils.

[29] Zhang R., Zhang Y., Song L., Song X., Hanninen H., Wu J. (2017). Biochar enhances nut quality of *Torreya grandis* and soil fertility under simulated nitrogen deposition. For. Ecol. Manag..

[30] Wang J.Y., Xiong Z.Q., Kuzyakov Y. (2016). Biochar stability in soil: Meta-analysis of decomposition and priming effects. Glob. Chang. Biol. Bioenergy.

[31] Lahori A.H., Zhanyu G., Zhang Z., Li R., Mahar A., Awasthi M. (2017). Use of biochar as an amendment for remediation of heavy metal-contaminated soils: Prospects and challenges. Pedosphere.

[32] Waqas M., Kim Y.H., Khan A.L., Shahzad R., Asaf S., Hamayun M. (2017). Additive effects due to biochar and endophyte application enable soybean to enhance nutrient uptake and modulate nutritional parameters. J. Zhejiang Univ.-Sci..

[33] Chen Y., Li R., Li B., Meng L. (2019). Biochar applications decrease reproductive potential of the English grain aphid *Sitobion avenae* and upregulate defense-related gene expression. Pest Manag. Sci..

[34] Fu Q., Baoping L., Ling M. (2018). Effects of biochar amendment to soil on life history traits of *Laodelphax striatellus* (Hemiptera: Delphacidae) on rice plants. Chin. J. Rice Sci..

[35] Cook S.P., Neto V.R.A. (2018). Laboratory Evaluation of the Direct Impact of Biochar on Adult Survival of Four Forest Insect Species. Northwest Sci..

[36] Hou X., Meng L., Li L., Pan G., Li B. (2015). Biochar amendment to soils impairs developmental and reproductive performances of a major rice pest *Nilaparvata lugens* (Homopera: Delphacidae). J. Appl. Entomol..

[37] Anderson N., Jones J.G., Page-Dumroese D., McCollum D., Baker S., Loeffler D., Chung W. (2013). A comparison of producer gas, biochar, and activated carbon from two distributed scale thermochemical conversion systems used to process forest biomass. Energies.

[38] SAS Institute, Inc. (2013). SAS/STAT^®^ 13.1 User’s Guide.

[39] Larson S. (1989). Stressful times for the plant stress-insect performance hypothesis. Oikos.

[40] Raubenheimer D., Simpson S.J. (1993). The geometry of compensatory feeding in the locust. Anim. Behav..

